# Editorial: The formal and informal workforce for a global aging population

**DOI:** 10.3389/fpubh.2026.1820846

**Published:** 2026-04-02

**Authors:** Colette J. Browning, Connie J. Evashwick, Shane A. Thomas

**Affiliations:** 1 Health Innovation and Transformation Centre, Federation University, Ballarat, VIC, Australia; 2 Institute of Health and Wellbeing, Federation University, Ballarat, VIC, Australia; 3 School of Public Health, San Diego State University, San Diego, CA, United States

**Keywords:** aged care, formal carers, health professionals, informal carers, population aging, workforce distribution, workforce training, geriatrics

## Introduction

The increased demand for formal and informal care for the large and rapidly growing aging population is a major problem globally. Although the aging population percentages vary across countries, many regions will see a doubling of people aged 65 and over between 2012 and 2050 (1). Population aging has major consequences for the informal and formal care of older people. As we have seen in a recent related Research Topic, serious economic consequences for individuals and governments are associated with population aging (2). Fields as diverse as housing, transportation, social services, retirement investing, and information technology are affected by this demographic trend. The articles in this collection are concerned with the health and related needs of older adults. To fulfill the WHO goals for healthy aging requires a multidisciplinary health and social care workforce with formal training at different levels of expertise (3). Moreover, the formal paid workforce must effectively collaborate with informal unpaid caregivers, including friends and family, to provide affordable, accessible, person-centered, and high-quality care.

The care needs of older people are often driven by poor chronic disease prevention and management and by multimorbidity (4). As well as increased total numbers of the health care workforce, the workforce must be skilled in the prevention and management of chronic illnesses (5) and sensitive to the special physical and psychological needs of older adults. The workforce must also be distributed geographically to give access to older adults across all geographical locations.

The purpose of this Research Topic was to bring together the latest data, current analyses, and innovative models related to the formal and informal workforce caring for older adults. The goal was to provoke thinking about what could be done by educators, community and health organizations, social service agencies, policy makers, professional associations, licensing bureaus, and healthcare professionals themselves, to maximize the capacity and competency of the future healthcare workforce to serve a global aging population.

## Highlights of the contributions to this Research Topic

This Research Topic includes 21 articles submitted from 94 authors from a range of countries, including Australia (8), China (5), USA (2) and one each from Ghana, Germany, Singapore, Malaysia, South Korea and Europe, reflecting the global nature of the workforce issues in a period of rapid population aging. The articles use a range of methodologies to interrogate the issues. These include evaluation studies, scoping reviews, qualitative interviews, quantitative surveys, analyses of secondary data, and a Delphi study. The main topics were categorized into four areas: supply of the formal and informal workforces; the training and education of carers; immigration and the care workforce; and the challenges faced by both the formal and informal workforce in providing high quality care.

## Supply of the formal and informal workforce

Five articles address the fundamental issue of the supply of the formal and informal workforce to care for the growing number of older adults. Three articles (Thomas et al., submitted^1^; Haß et al.; Boye et al.) focus on the numbers and geographic distribution of healthcare professionals. Two articles (Blackberry et al.) and (Huang et al.) examine the economics of caregiving and what must be done to make caregiving financially viable for institutions, governments, and individuals.

Haß et al. projected the number of care-dependent people in Germany through 2050 as a basis for the nation to develop strategies to meet the demand. Using eight different scenarios, they used mathematical models to estimate a growth in the number of care- dependent people from between 5.6 million to as many as 14 million. They concluded that the projections lead to potential economic challenges as well as stronger demand for healthcare and nursing personnel.

Rural communities in many countries experience a gap between the availability of health care professionals and the needs of their aging communities (6). Thomas et al., submitted (see text footnote 1) used mapping to visually display the mismatch between the aged population and health professionals. They argued that while increasing the numbers of health professionals in rural communities is important, their clinical skills must match the needs of older people, particularly those with complex care needs and multi-morbidities.

An e-Delphi study conducted with doctors and directors of health services in Ghana found that financial incentives, access to professional development opportunities, and good schools for children were key drivers of willingness to work in rural settings (Boye et al.).

Blackberry et al. addressed issues in the care economy, formal and informal support, across all life stages and abilities. Twenty-five percent of care was situated in aged care, and spouses provided most of the informal support. Barriers to care included psychological stress and cost. Women were impacted most in terms of loss of income in their carer role.

In an analysis of the performance of Community Care Facilities in China, Huang et al. concluded that the shortage of skilled aged care workers, lack of needs-based planning for their location, and poor promotion of the services available in these facilities to older people were important problems.

## Formal and informal workforce training and education

Four articles address the training needs of formal and informal carers (Bressington et al.; Rayner et al.; Reinschmidt et al.; Jing et al.). Health professionals and aged care workers may or may not receive training as part of their professional training and ongoing professional development. In contrast, informal carers, such as spouses or relatives, often do not have training opportunities.

Bressington et al. conducted a narrative synthesis to investigate the type and efficacy of online training for informal (unpaid) carers. The efficacy of online training was found to be greatest for carers of people with dementia and carers of people with mental health conditions. The authors recommended using co-design training approaches in future studies to improve participants' engagement and relevant content.

Personal Care Workers (PCWs) make up most of the workforce in nursing homes in many countries. Rayner et al. reported the design, development and implementation of training packages for PCWs in Australia. The researchers incorporated self-directed learning and the recognition of existing skills to embed the training in real world situations. A train-the-trainer approach was used, and reach was increased through on-line education. Similarly, in the US, Reinschmidt et al. focused on the effectiveness of a train-the-trainer model for dementia training for Community Health Workers who were predominantly of American Indian or Alaskan Native background. Knowledge and self-efficacy improved, and at follow-up, participants were effectively using the course information in their practice. In a scoping review, Jing et al. highlighted the positive effects of community-based education, where students interact with older adults, in improving empathy, emotional intelligence, and caring behaviors in nursing students.

## Implications of migration on care workers and recipients

Migrants make up a large percentage of the carer workforce in many countries (7).

Four articles addressed issues related to migrant and refugee care workers and the care needs of immigrants (Finco et al.; Winarnita et al.; Watts; Xu et al.).

Through SWOT analysis, Finco et al. evaluated an aged care course for migrants and refugees conducted in Cyprus, Greece, Italy, and Portugal. Study participants included course participants and experts in aged care education. Barriers to participation included costs and lack of education. Practical rather than theoretical knowledge was endorsed, while on-line delivery methods were not favored.

Australia is a multicultural nation where a large percentage of the aged care workforce are migrants from various countries. In residential aged care (nursing homes), 35% of employees are from culturally diverse backgrounds (8) and are an important growing resource in an industry where a workforce shortfall of 110,000 is predicted in the next 10 years (9). Understanding issues around attracting and retaining the aged care workforce is important to address this potential shortfall. Winarnita et al. studied the lived experiences of Asian female migrant workers in Australia using the concepts of agency and institutional structures to understand the role of social resilience and its relationship to access and retention of this workforce. The authors concluded that social resilience built through social support networks, including workforce supports, can contribute to career trajectories and retention and requires management approaches and training opportunities. Watts provided a narrative perspective on the need for an Afro-centric workforce to support older African-born Australians, who are increasing in number. She highlighted the cultural practices common in African communities, including respect, connection to kin, and community connections often through co-residency. While the aged care workforce in Australia incudes the African Australian diaspora, Watts proposes measures to increase that workforce by training aged care workers and health professional in African cultural norms, highlighting role models in the field, targeting of scholarships to the African community, and matching older adults with kin through volunteer programs.

Xu et al. addressed the match of cultural understanding between carers and recipients from the opposite perspective. They conducted a scoping review of qualitative studies investigating the needs of older Chinese who have immigrated to Europe. They found a robust literature describing the challenges older Chinese immigrants living in Europe face in meeting social and health needs because of differences in values, language, attitudes toward care, and expectations about the role of younger generation family members as carers.

## Challenges of providing adequate care

Eight articles investigated the challenges the workforce faces in providing care (Alsaeed et al.; Zainal et al.; Cheng et al.; Kim et al.; Liu et al.; Hu et al.; Fan et al.; Thomas Hebdon et al.). These range from factors in the organizational environment to factors that cause individuals stress. Specifically addressed were the adoption of digital health technologies (Alsaeed et al.; Zainal et al.), concerns by informal carers about their expertise in providing care (Cheng et al.), professional identity and job stress (Kim et al.), the adequacy of nurses' knowledge, attitudes and practice in aged care (Liu et al.), and stress and mental health issues in caregivers (Hu et al.; Fan et al.; Thomas Hebdon et al.).

Kim et al. found that higher job stress, higher education level, and lower professional self-concept predicted staff turnover in nursing staff working in dementia centers in Korea. In a qualitative study conducted in China, Cheng et al. interviewed participants who were caring for a person with dementia. Carers experienced mental health issues related to poor social support and financial strain, as well as issues with effective communication with people with dementia. Lack of professional training in dementia care was an issue for both formal and informal carers.

Nurses working in hospice care showed limited knowledge of hospice care methods and regulations, low confidence in performing their duties, and although providing good psychological support, had poor skills in case management (Liu et al.). A cross-sectional survey of caregivers in nursing homes in China found high levels of depression and stress, particularly in rural settings and where caregivers experienced chronic illnesses (Fan et al.).

An emerging issue in aged care is the use of digital health and support technologies in the care of older people. Alsaeed et al. highlighted the need to address technology adoption in aged care through its use in care planning and medication, robotic pets for support and companionship, touch-screen devices for communication, and virtual reality for entertainment and reminiscence. Many health and aged care services now employ telehealth consultations and digital reminder systems in service design. A qualitative study in Singapore explored the challenges faced by informal caregivers of integrating digital health technologies in their care of older people (Zainal et al.). Barriers highlighted included digital literacy gaps and training in the use of technologies, affordability, slow responsiveness of health care providers, and inadequacies in telehealth consultations for people with complex needs. This study highlights the need for age-friendly affordable digital health technologies and accessible training opportunities for carers.

Thomas Hebdon et al. used semi-structured interviews to investigate the experiences of Millennial (those born between 1981 and 1996) family caregivers of people with a disability or chronic illness in the US. Millennials are part of the “sandwich generation,” as they may have joint responsibilities in caring for their children and parents (10). Many of the care receivers in this study were adults with age related illnesses such as dementia and cancer. Difficulties concerning balancing work and caregiving roles leading to financial strain were highlighted. Latinos in the study sample faced additional stressors including racism, migrant status, and health care access.

In a scoping review, Hu et al. found that grandparenting could be emotionally rewarding or a stressor depending on the context of the caregiving but that various research gaps included methodological biases, measurement issues, and insufficient consideration of mediating and moderating variables such as childhood characteristics or social support.

## Significance and impact

The studies included in this Research Topic provided a global view of issues regarding the formal and informal workforces who will care for an increasing number of older people around the world. Despite years of emphasis on the need for geriatric specialists in all disciplines of healthcare professions and the need to support family, friends, and informal caregivers, models of success remain elusive. The number of healthcare practitioners specializing in aging appears insufficient in any country to meet the healthcare needs of older adults. Moreover, only a couple of articles submitted to this Research Topic indicated that any country is working on projections with the intent of finding ways to meet the demand. Contrary to our expectation, education of geriatric specialists in medicine, nursing, dentistry, physical therapy, or other critical disciplines was not addressed by any research reported in this Research Topic. Education of informal caregivers received attention, but despite several scoping reviews, more challenges than solutions were identified by the contributing researchers. While improved education was acknowledged as a key requirement for both informal and formal carers, limited detail was provided about how this might be structured and funded.

Depending upon immigration to solve the shortage in supply of carers across nations is fraught with problems, one of the most prominent being the mismatch between cultural and linguistic backgrounds of those providing and those receiving care. Caregiver stress remains a well-recognized but unsolved problem. At least the articles included here indicate that several approaches are being tried and that researchers are looking for evidence-based programs that work.

The WHO Health Workforce Support and Safeguards List provides statistical updates to global health workforce shortfalls, current to 2026, in which it notes 55 countries with serious health workforce shortfalls (11). It recommends against recruitment in these countries to transfer practitioners to other better resourced countries. A review in the Milbank Quarterly (12) also provides a comprehensive overview of global policies regarding formal and informal health workers and similarly concludes that there is a significant global under-provision of workers that requires increased investment and policy focus. Most countries have health workforce policies that aspire to increased formal and informal health care workforces. In the US changes to immigration policies have created concerns because of the predicted impacts upon the US care workforce which relies heavily on immigration (13). The Australian National Medical Workforce Strategy (14) points to the same global concerns shared by most countries. Australian policy supports its 2.65 million informal carers through the National Carer Strategy 2024–2034 (15), which is an important complement to its formal staffing policies. The UK has its NHS Long Term Workforce Plan which targets workforce increases by 431,000 by 2036 (16). The UK also supports informal carers through legislation in mandatory annual leave entitlements. Many countries have responded to the seemingly universal informal and formal care workforce shortages but making headway in a situation of global shortages is challenging.

Finally, the economic underpinning to support the potentially overwhelming demand for care for older adults is a concern of individuals, institutions, and governments—but little research is directed at delineating costs and benefits of chronic care or finding solutions that will resolve national and cross-national conditions to ensure the health of older people.

## Future directions

A greater range and more in-depth data collected about this phenomenon of caring for older adults and better evidence concerning the efficacy of programs to address it are imperative. At a national level, more sophisticated health economics analyses of the costs and benefits of care for older people would be desirable for informing public policy development. Universities and local colleges could examine their curricula and degree offerings to offer more education for the formal healthcare workforce, but techniques must be found to motivate institutions and individual faculty to increase the content of geriatrics in their curricula. Continued emphasis on cultural sensitivity is an essential component of the training for all those who work in healthcare, whether in the formal or informal workforce, as professionals or family members and friends. The support programs for informal caregivers and those who provide basic care for functional assistance seem tailored to specific communities and cultures; perhaps this principle is fundamental to success. The articles in this Research Topic, individually and collectively, contribute to understanding the state of knowledge about the workforce that cares for older adults worldwide. They also reveal how much more remains to be considered, studied, and discovered.

## Footnotes

1 Thomas SA, Evashwick CJ, Nguyen H, Young AE, Charchar F, Prestes P, et al. Meeting the health needs of an aging population: health workforce distribution and training across Australia. *Front Public Health*. (Manuscript submitted).
